# Experimental Study on Thermal Conductivity and Hardness of Cu and Ni Nanoparticle Packed Bed for Thermoelectric Application

**DOI:** 10.1186/s11671-017-1969-0

**Published:** 2017-03-11

**Authors:** Zi-Zhen Lin, Cong-Liang Huang, Wen-Kai Zhen, Yan-Hui Feng, Xin-Xin Zhang, Ge Wang

**Affiliations:** 1 School of Electrical and Power Engineering, China University of Mining and Technology, Xuzhou, 221116 China; 2 School of Mechanical Engineering, University of Science and Technology Beijing, Beijing, 100083 China; 3 School of Materials Science and Engineering, University of Science and Technology Beijing, Beijing, 100083 China

**Keywords:** Thermal conductivity, Thermoelectric materials, Nanoparticle packed bed, Nanoporous material, Vickers hardness

## Abstract

The hot-wire method is applied in this paper to probe the thermal conductivity (TC) of Cu and Ni nanoparticle packed beds (NPBs). A different decrease tendency of TC versus porosity than that currently known is discovered. The relationship between the porosity and nanostructure is investigated to explain this unusual phenomenon. It is found that the porosity dominates the TC of the NPB in large porosities, while the TC depends on the contact area between nanoparticles in small porosities. Meanwhile, the Vickers hardness (HV) of NPBs is also measured. It turns out that the enlarged contact area between nanoparticles is responsible for the rapid increase of HV in large porosity, and the saturated nanoparticle deformation is responsible for the small increase of HV in low porosity. With both TC and HV considered, it can be pointed out that a structure of NPB with a porosity of 0.25 is preferable as a thermoelectric material because of the low TC and the higher hardness. Although Cu and Ni are not good thermoelectric materials, this study is supposed to provide an effective way to optimize thermoelectric figure of merit (ZT) and HV of nanoporous materials prepared by the cold-pressing method.

## Background

For thermoelectric materials, the efficiency of thermoelectric energy conversion can be described by the materials’ thermoelectric figure of merit (ZT)*,* which can be defined as ZT = *S* ^2^ *σT*/(*k*_*p*_ + *k*_*e*_ + *k*_bipolar_) [1], where *S*, *σ*, *T*, *k*_*p*_, *k*_*e*_, and *k*_bipolar_ are the Seebeck coefficient, electrical conductivity (EC), temperature, and the lattice, electronic, and bipolar parts of the thermal conductivity (TC), respectively. ZT is mainly determined by the materials’ effective TC, EC, temperature, and Seebeck coefficient. A good thermoelectric material should possess high *S* to achieve high voltage output, high *σ* to reduce Joule heat loss, and low *k* (effective TC) to maintain a large temperature difference. A normal way to optimize ZT of a material is to increase the power factor *S* ^2^ *σ* by optimizing the carrier concentration and to reduce *k*_*p*_ by introducing scatter centers to the phonons. In order to improve the thermoelectric materials’ ZT, the method of increasing *σ* and/or *S* and/or decreasing *k* by tuning the structure of thermoelectric materials is widely applied. The reduction of TC sometimes also leads to a decrease of EC, contrary to our goal to decrease TC and increase EC at the same time. To minimize the contradiction, nanoporous materials have drawn wide attention [2]. Nanoporous materials with high density of interfaces offer an alternative to superlattices as potential thermoelectric materials [3], because the presence of closed or connected pores in porous materials provides an effective scattering mechanism for mid- and long-wavelength phonons that contribute heavily to TC [4]. So, the nanoporous materials are not only potential thermoelectric materials, but also high-performance insulation materials [5, 6]. In spite of the potential applications, the study of the thermal physical properties of nanoporous materials is also valuable for understanding the underlying mechanism. In this paper, we focus on the TC and hardness of a kind of nanoporous material to illustrate the potential advantage of nanoporous materials for thermoelectric application.

There are already some methods to prepare nanoporous materials, for example, the dealloying method [7–10], organics pyrolytic method [11, 12], sol-gel method [13–15], etc. While these methods are usually applied to prepare isotropic nanoporous materials, there is no interface existing in these nanoporous materials which is preferable for getting a low TC. For introducing interfaces into nanoporous materials, the nanoparticle tableting method [16–21] is applied to prepare the nanoporous materials in this paper. A force is usually applied to press the nanoparticles into large-scale nanoporous materials in the tableting method. There are three different ways to prepare nanoparticle tablets [22–28]: the hot-pressing method, the cold-pressing method, and the self-forming method. Different methods will lead to different porosities. The porosity of a nanoparticle powder can be larger than 85% in the absence of the pressing method applied [29, 30]. For the hot-pressing method, the porosity of the nanoparticle tablet can be lower than 10% [31], while the porosity could span a large scale for the cold-pressing method. Nanoporous tablet which is composed of air and nanoparticles is also called a nanoparticle packed bed (NPB). The cold-pressing method is applied in this paper to obtain a large span of porosity, which ranges from 15 to 40%.

In this paper, with the Cu and Ni nanoparticles pressed into NPBs, respectively, the relationship between the tableting pressure and the porosity is investigated to indicate the connection between the porosity and the nanostructure. Then, the TC of NPBs at low porosities (15–40%) are experimentally studied, resulting in the discovery of a different decreasing tendency of TC versus porosity than that currently known, which is explained herein. Meanwhile, the Vickers hardness (HV) of the NPBs at different porosities is also measured to assess the mechanical properties of NPBs additionally. Finally, the TC and the HV are both discussed for figuring out a proper porosity which is suitable for thermoelectric application.

## Methods

**Fig. 1:**
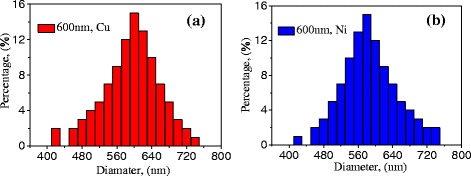
**a** Cu nanoparticles with a diameter of 600 nm; **b** Ni nanoparticles with a diameter of 600 nm

**Fig. 2:**
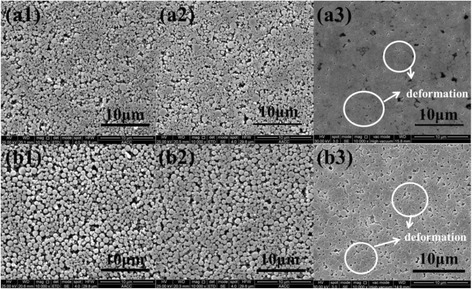
Microstructure of Cu and Ni NPBs. **a1** Cu with 18 MPa. **a2** Cu with 24 MPa. **a3** Cu with 32 MPa. **b1** Ni with 18 MPa. **b2** Ni with 24 MPa. **b3** Ni with 32 MPa

Cu and Ni nanoparticles with diameters of 600 nm are commercially obtained from Beijing DK Nano Technology Co., Ltd. The nanoparticles are first dispersed by ultrasonic waves; meanwhile, laser nanoparticle analyzer is applied to the analysis of the nanoparticle size distributions. Results are shown in Fig. 1. Then, nanoparticles are pressed into NPBs with the cold-pressing method at room temperature. Ten different stamping pressures (14, 16, 18, 20, 22, 24, 26, 28, 30, and 32 MPa) are applied to obtain different NPBs. For obtaining an average physical property, five NPBs are made at each pressure. Microstructures of NPBs stamped with different pressures (18, 24, and 32 MPa) are shown in Fig. 2, observed with field-emission scanning electron microscopy (SEM) on a QuantaTM-250 instrument (FEI Co., USA) with accelerating voltage of 30 or 25 kV. It shows that nanoparticles in NPBs are evenly distributed. The nanoparticles in Fig. 2a1, a2, b1, and b2 remain in their original shape when low pressure is applied. When high pressure is applied, nanoparticle deformation occurs, as shown in Fig. 2a3, b3.

**Fig. 3:**
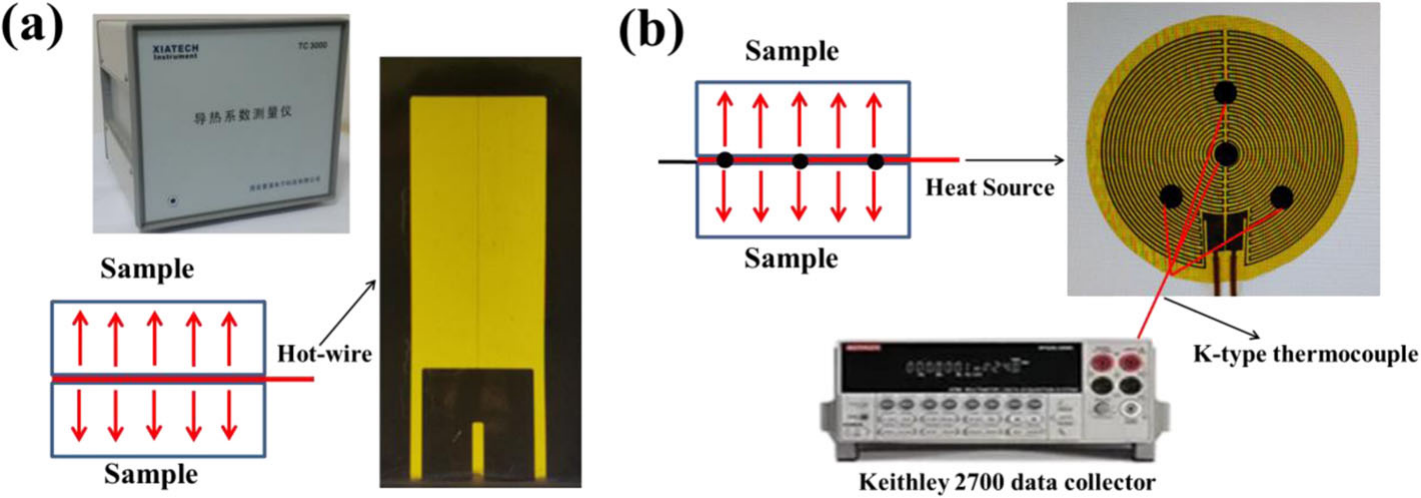
Thermal conductivity measurement equipment. **a** Hot-wire method. **b** Transient plane heat source method

The TC of NPBs is measured using the hot-wire method; meanwhile, the transient plane source (TPS) [32–34] method is applied to test the hot-wire results. The hot-wire measurement is carried out with a commercial device (Model TC3000, Xian XIATECH Technology Co.), as shown in Fig. 3a. A platinum wire with a diameter of 76.2 μm serves both as the heater and the electrical resistance thermometer. The surface of the platinum wire is coated with a thin electrical insulation epoxy to prevent short circuiting. The temperature variation is obtained by measuring the change of electrical resistance versus time; with the temperature versus time obtained at a constant electric heating power, the TC can be easily determined [29] with an instrumental error estimated to be less than 3%. With the hot wire sandwiched between two pieces of the same sample, as shown in Fig. 3a, measured along five different directions separately, a mean TC is obtained by averaging the TC along different directions, and the deviation of the TC is less than 6.5%. The TPS experimental device is shown in Fig. 3b. The heating plane consists of an electrical conducting pattern of thin Constantan wire (0.1 mm diameter) in the form of a spiral, inserted between two insulating layers of polyimide, as shown in Fig. 3b, where the polyimide is used as protection and electrical insulation. The heating plane is sandwiched between two pieces of the same sample. Eight K-type thermocouples with a diameter of 0.1 mm are applied as the temperature sensor. A data collector (Model 2700, Keithley Co., USA) is used to collect the temperature data. With about 200 temperature points obtained, the relation between temperature and time can be established. The recorded data during the first few seconds are omitted to reduce the initial thermocouple thermal-mass influence. With four sensors set on each side of the plane, eight TCs can be calculated at each measurement, and then a mean TC is calculated.

A Tukon™ 1102/1202 Vickers hardness tester is applied to measure the HV hardness. In the experiment, a force of 9.8 N is applied to press the diamond probe into the samples, and the force is maintained for 15 s. Finally, the HV of the materials can be reflected by the size of the surface indentation. To obtain the average hardness, five different locations on each NPB are selected to measure the HV. The relative error of the HV is less than 8.3%. The randomness of the testing locations should be responsible for this deviation.

## Results and Discussions

To reveal the relationship between TC and nanostructures of NPBs, we firstly probe the influence of pressing pressure on the porosity of NPBs. Then, the TC and the HV at different porosities are discussed. Finally, with the thermal and mechanical properties considered, the most suitable porosity in NPBs is indicated for thermoelectric application.

**Fig. 4:**
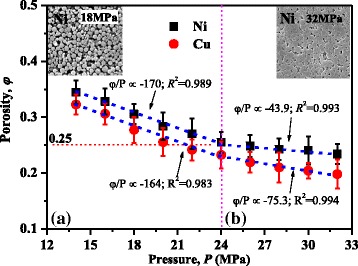
The inlet shows the microstructure of Ni NPBs

In the NPBs preparation, with increasing pressure, different contact conditions between nanoparticles will lead to different decreasing tendencies of porosities. In order to find out the relation between the porosity and the contact conditions, the porosity versus pressing pressure is shown in Fig. 4. There are two different decreasing tendencies of porosities with increasing pressure. According to the different slope of two tendencies, the pressing process is divided into processes A and B. In process A, the loaded pressure makes nanoparticles close to each other, and only few nanoparticle extrusion deformations occur, as that shown in the inlet of Fig. 4. This is also confirmed by the fact that a same porosity-decreasing slope is observed for different materials in process A. The porosity of NPBs decreases linearly with increasing pressure until it reaches a porosity of about 0.25 for both Cu and Ni. With a larger pressure loaded, it will be process B. In process B, obvious nanoparticle extrusion deformations occur, which can be seen in the inset of Fig. 4. The nanoparticle extrusion deformation dominates in process B, and the porosity decreases much more slowly than that in process A. The different decrease slope in process B is attributed to the different hardness of Cu and Ni (the HV is 125 for bulk Cu and 160 for bulk Ni [35]).

**Fig. 5:**
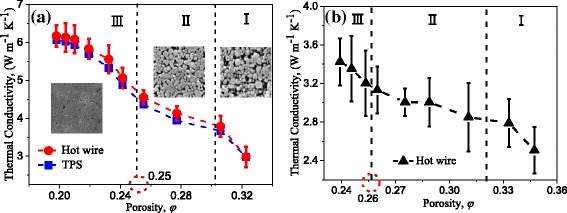
Thermal conductivity at different porosities. **a** Cu NPBs. **b** Ni NPBs

After the relationship between porosity and nanostructures was established, the TC of Cu and Ni NPBs with different porosities are studied and shown in Fig. 5. The results obtained with the hot-wire and the TPS methods are compared in Fig. 5a. It indicates that the TC difference between these two different methods will be less than 5%. It confirms that the result obtained with the hot-wire method is reliable in this paper. As expected, the TC of NPBs is less than 2% that of the bulk, which correspond to 397 W m^−1^ K^−1^ for Cu [36]. The pore and the interface between nanoparticles, which will cause an additional thermal resistance, should be responsible for the low thermal conductivity [4, 37, 38]. Moreover, there is a threefold TC decrease tendency in the whole porosity span, as shown in Fig. 5, which is beyond the scope of our knowledge. This should be caused by different contact conditions between nanoparticles at different porosities. This phenomenon is thoroughly discussed in the next part.

For convenience, the tendency is divided into three parts denoted as parts I, II, and III, respectively. In part I (*φ* ≥ 0.3), the TC increases rapidly with decreasing porosity. This is caused by the increase of heat-transfer routes due to the increases of particle contacts. In part II (0.26 < *φ* < 0.30), the increase of thermal conductivity with decreasing porosity is much lower than that in part I. This is thought to be caused by the inhomogeneous porosity in NPBs. With a larger pressure loaded in part II, extrusion deformation emerges among some nanoparticles. Although a larger pressure will lead to more extrusion deformation among nanoparticles, there are still some nanoparticles that remain unchanged in the inner part of NPBs. The large thermal resistance in these unchanged separated nanoparticles will dominate the resistance. Therefore, if there are still some nanoparticles that have not been deformed, the effective TC will not be obviously increased. At the end of part II, extrusion deformation has happened for most of the nanoparticles. With a larger pressure loaded, severe deformation will occur in part III (this can be seen from the SEM image in Fig. 5a). A severe deformation will lead to a larger contact interface. With an increase of the area of the contact interface, the TC of Cu NPBs increases rapidly. At the end of the part III, the TC tends to be a constant. Similar tendencies of TC-versus-porosity are also observed for Ni NPBs in Fig. 5b, which confirms the above discussed result about Cu NPBs. In conclusion, the porosity dominates the TC of the NPBs in part I while the inhomogeneous porosity dominates in part II, and the TC depends on the contact area between nanoparticles in part III.

**Fig. 6:**
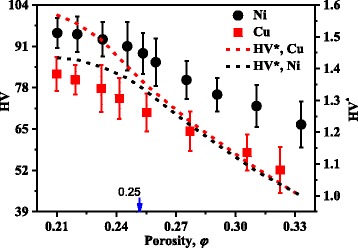
Vickers hardness (HV) of Cu and Ni NPBs with different porosities

The hardness of Cu and Ni NPBs is discussed in this part. The HV and the dimensionless HV*, which is calculated by $\mathrm{H}\mathrm{I}\mathrm{V}*=\frac{\mathrm{HI}\mathrm{V}}{\mathrm{HI}{\mathrm{V}}_{\varphi =0.33}}$, are shown in Fig. 6. It shows that the hardness of Cu (Ni) NPBs prepared with 32 Mpa tableting pressure is about 1.56 times (1.43 times) that of 14 Mpa at room temperature. While the porosity of NPBs becomes smaller than 0.25, the HV of both Cu and Ni NPBs tends to be a constant. The enlarged contact area between nanoparticles is responsible for the rapid increase of HV at large porosity ranging from 0.33 to 0.25, while the saturated nanoparticle deformation is responsible for the small increase of HV at low porosities (smaller than 0.25). Actually, the change of HV reflects the structural transformation. For example, a large HV implies a good contact between different nanoparticles; it also means a higher TC because of a larger contact area. With the porosity much smaller than 0.25, the TC and the HV of NPBs will both tend to be constant values, which means a serious nanoparticle deformation in NPBs. Because the TC and the HV both depend on the nanostructure of NPBs, HV versus porosity in Fig. 5 follows almost a similar rule to that of TC. From the points of the low TC and the higher mechanical property, the porosity at the end of part II, it is 0.25 in this paper, may be preferable to develop a thermoelectric material. A lower porosity smaller than 0.25 will lead to a rapid increase of TC which is harmful for improving the performance of a thermoelectric material. For porosity *φ* = 0.25, the TC of Cu (Ni) NPBs is about 4.36 (3.13) W m^−1^ K^−1^, which is less than 1.1% (3.4%) that of the bulk, while the HV of Cu (Ni) NPBs is about 65.8% (56.8%) that of bulk Cu (Ni) where the HV is 125 for bulk Cu and 160 for bulk Ni [35]), which suggests that the NPBs are strong enough for application. Thus, 0.25 may be the most preferable porosity. Although Cu and Ni are not good thermoelectric materials, discussions in this paper should be also true for other materials, for which the preferable porosity may be different.

## Conclusions

A kind of nanoporous material which is a potential thermoelectric material is discussed in this paper. We try to probe a way to decrease the TC of NPBs and to enhance the hardness of NPBs at the same time. With nanostructures of NPB illustrated by SEM, the relationship between the pressing pressure and the porosity is discussed. The TC of NPBs is measured with a hot-wire method, and the TPS method is applied as a testing group. The porosity dominates the TC of the NPB in large porosities, while the TC depends on the contact area between nanoparticles in small porosities. The HV of NPBs at different porosities is also measured with a Vickers hardness tester. Results turn out that when the porosity of NPBs becomes smaller than 0.25, the HV tends to be a constant like what happens for TC. With both TC and HV considered, it can be pointed out that a structure of NPB with a porosity of 0.25 is preferable as a thermoelectric material in this paper, because of the low TC and high hardness. Although Cu and Ni are not good thermoelectric materials, the discussion in this paper should be also true for other materials, for which the proper porosity may be different.

## Nomenclature

*T* Temperature

NPB Nanoparticle packed bed

TC Thermal conductivity

HV Vickers hardness

ZT Thermoelectric figure of merit

## Symbols

*k* Effective thermal conductivity

*kp* Phonon thermal conductivity

*k*_*e*_ Electronic thermal conductivity

*k*_*bipolar*_ Bipolar thermal conductivity

*P* Pressure

*S* Seebeck coefficient

## Greek symbols

*ρ*_*1*_ Density of the NPB

*ρ*_*2*_ Density of the bulk

*φ* Porosity

*σ* Electric conductivity

## Subscripts

* Dimensionless quantity

*e* Electron

*p* Phonon
